# “Velcro-type” crackles predict specific radiologic features of fibrotic interstitial lung disease

**DOI:** 10.1186/s12890-018-0670-0

**Published:** 2018-06-18

**Authors:** Giacomo Sgalla, Simon L. F. Walsh, Nicola Sverzellati, Sophie Fletcher, Stefania Cerri, Borislav Dimitrov, Dragana Nikolic, Anna Barney, Fabrizio Pancaldi, Luca Larcher, Fabrizio Luppi, Mark G. Jones, Donna Davies, Luca Richeldi

**Affiliations:** 1 Division of Respiratory Medicine, University Hospital “A. Gemelli”, Catholic University of Sacred Heart, Rome, Italy; 2 King’s College Hospital, London, UK; 3 University Hospital of Parma, Parma, Italy; 4 National Institute for Health Research Southampton Respiratory Biomedical Research Unit and Clinical and Experimental Sciences, University of Southampton, Southampton, UK; 5 Centre for Rare Lung Disease, University Hospital of Modena, Modena, Italy; 6 Medical Statistics, Faculty of Medicine, University of Southampton, Southampton, UK; 7 Institute for Sound and Vibration Research, University of Southampton, Southampton, UK; 8 DISMI, University of Modena and Reggio Emilia, Reggio Emilia, Italy

**Keywords:** Fibrotic interstitial lung disease, Idiopathic pulmonary fibrosis, Velcro crackles, Lung sounds, Breath sounds

## Abstract

**Background:**

“Velcro-type” crackles on chest auscultation are considered a typical acoustic finding of Fibrotic Interstitial Lung Disease (FILD), however whether they may have a role in the early detection of these disorders has been unknown. This study investigated how “Velcro-type” crackles correlate with the presence of distinct patterns of FILD and individual radiologic features of pulmonary fibrosis on High Resolution Computed Tomography (HRCT).

**Methods:**

Lung sounds were digitally recorded from subjects immediately prior to undergoing clinically indicated chest HRCT. Audio files were independently assessed by two chest physicians and both full volume and single HRCT sections corresponding to the recording sites were extracted. The relationships between audible “Velcro-type” crackles and radiologic HRCT patterns and individual features of pulmonary fibrosis were investigated using multivariate regression models.

**Results:**

148 subjects were enrolled: bilateral “Velcro-type” crackles predicted the presence of FILD at HRCT (OR 13.46, 95% CI 5.85–30.96, *p* < 0.001) and most strongly the Usual Interstitial Pneumonia (UIP) pattern (OR 19.8, 95% CI 5.28–74.25, p < 0.001). Extent of isolated reticulation (OR 2.04, 95% CI 1.62–2.57, p < 0.001), honeycombing (OR 1.88, 95% CI 1.24–2.83, < 0.01), ground glass opacities (OR 1.74, 95% CI 1.29–2.32, p < 0.001) and traction bronchiectasis (OR 1.55, 95% CI 1.03–2.32, *p* < 0.05) were all independently associated with the presence of “Velcro-type” crackles.

**Conclusions:**

“Velcro-type” crackles predict the presence of FILD and directly correlate with the extent of distinct radiologic features of pulmonary fibrosis. Such evidence provides grounds for further investigation of lung sounds as an early identification tool in FILD.

## Background

Fibrotic interstitial lung disease (FILD) represents a diverse and challenging group of disorders with varied treatment strategies and prognoses. Idiopathic pulmonary fibrosis (IPF) is the most frequent and deadly among FILD [1]. Recognition of signs and symptoms suggestive of FILD by healthcare practitioners represents a valuable point-of-care, low-cost opportunity for detection of early disease and timely diagnostic work up [2–4]. Chest auscultation remains an important clinical assessment in patients with respiratory disorders, providing immediate and reliable information to clinicians [5]. “Velcro-type” crackles are brief, discontinuous pathological lung sounds, explosive and transient in character, named after their similarity to the sound generated by Velcro strips separating [6]. Historically, “Velcro-type” crackles have been considered representative of established lung fibrosis [7], and quantitative analysis of crackles in ILD confirmed distinctive features as compared to those generated in other disorders such as chronic heart failure and pneumonia [8, 9]. While international consensus guidelines recommend that IPF is suspected in all patients with bibasilar inspiratory “Velcro-type” crackles [10], the direct association between “Velcro-type” crackles and specific radiologic features of pulmonary fibrosis has not been thoroughly clarified.

In this prospective case-control study, we systematically investigate the relationships between audible digitally recorded “Velcro-type” crackles and HRCT features and patterns of FILD with the aim of providing substantial evidence as to the potential role of lung sounds as a screening and monitoring tool in ILD. Lung sounds were digitally recorded from subjects immediately prior to undergoing clinically indicated HRCT. Audio files were independently assessed by two chest physicians and both full volume and single HRCT sections corresponding to the recording sites were extracted. The relationship between audible “Velcro-type” crackles and radiologic HRCT patterns or individual features of pulmonary fibrosis was investigated using multivariate regression models.

## Methods

### Study population and data collection

A total of 254 subjects referred to undergo HRCT scan of the chest for various clinical indications were consecutively recruited at the Radiology Units of the University Hospitals of Modena and Parma, Italy, between January 2013 and February 2015. Patients were considered eligible if they were aged 18 years or over with capacity to provide valid informed consent.

**Fig. 1:**
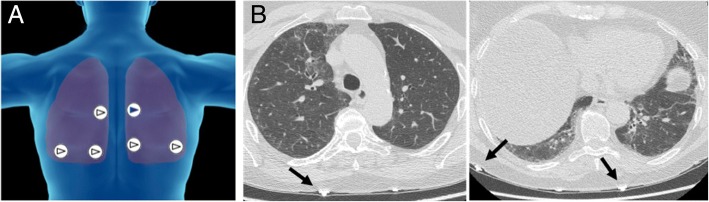
Recording sites selected in the study. Per each side of the chest, two recordings were performed at the lung bases at seven cm below the scapular angle, at both two and five cm from the paravertebral line respectively; another recording was taken from mid chest in correspondence of the fourth or fifth intercostal space, at two cm from the paravertebral line (**a**). Metallic marks were applied to the posterior chest of the patient and were visible at HRCT (black arrows, (**b**)

Demographics, smoking history and family history for respiratory disorders were collected. The clinical indication for performing the HRCT scan was also collected when available as reported on slips or letters from the general practitioner or respiratory consultants. However, since patients were referred from several different physicians and centres, it was not feasible to collect further data from the following diagnostic workup. Just prior to the HRCT, lung sounds were recorded sequentially at six anatomical sites identified, based on the guidelines for Computerized Respiratory Sounds Analysis (CORSA) [11], on the posterior chest as indicated in Fig. 1a. Sounds were recorded for ten seconds at each site, a time sufficient to record a minimum of two full breathing cycles, using an electronic stethoscope (Littmann 3200, 3 M, USA). After each recording, a small, radio-opaque metallic mark (a bio-compatible electrocardiography electrode) was applied to the skin to allow visualization and hence correlation of the recording sites on the HRCT (Fig. 1b). The recordings were transferred to the Littmann StethAssist software (3 M, USA) via Bluetooth technology, and exported in the wav format (sampled at 4 kHz with a resolution of 16-bit). The study was approved by the local ethics committee of Modena and Parma (Italy). Written informed consent was collected from all participants.

### Radiologic review

Full volume scans and single HRCT sections corresponding to the visible marked sites of recording were extracted for each study participant. After randomization, single HRCT sections were blindly reviewed by two other thoracic radiologists (D.M.H., and N.S.) with 28 and 12 years’ experience and semi-quantitatively scored on 3 different features: 1) the presence or absence of pulmonary fibrosis 2) HRCT patterns of reticulation, honeycombing, ground glass and emphysema, as defined in the Fleischner society glossary of thoracic imaging [12] and 3) severity of traction bronchiectasis. This semi-quantitative scoring system was similar to those used in previous studies [13, 14]. For reticulation, honeycombing, ground glass and emphysema the scores were defined by the proportion of bronchopulmonary segments involved (absence = 0; ≤ 25% = 1; > 25% and ≤ 50% = 2; > 50 and < 75% =3; ≥ 75% =4). Traction bronchiectasis was assigned with a categorical “severity” score (none = 0, mild = 1, moderate = 2, severe = 3) that accounted for the average degree of airway dilatation within areas of fibrosis as well as the extent of traction bronchiectasis throughout the lobe. Qualitative scores for pulmonary fibrosis were combined by an independent researcher (G.S.), who adjudicated an image as fibrotic in case of disagreement between the two observers. The semi-quantitative scores for individual features were averaged between the two observers. 76 Cases were identified based on the evidence of pulmonary fibrosis at one of the HRCT sections. 72 age and sex-matched controls were then selected from the remaining subjects showing no signs of fibrosis on the HRCT. As such, 148 subjects formed the final study population.

An expert thoracic radiologist (S.L.F.W.) with 10 years’ experience blindly reviewed the full volume HRCT scans of cases and controls for the radiologic evidence of a diffuse fibrosing lung disease. Scans adjudicated as FILD were further classified as Usual Interstitial Pneumonia (UIP), possible UIP or inconsistent with UIP according to validated radiologic criteria [15].

### Assessment of “Velcro-type” crackles

Two expert ILD physicians, blind to the clinical and radiology data, qualitatively assessed sound recordings for the presence of “Velcro-type” crackles. The sound files were played via personal computer using an open-source tool (Audacity software) and over-ear headphones (Sennheiser HD201 closed dynamic stereo). The sound files were randomized prior to assessment to avoid all files from the same subject being assessed consecutively. An independent researcher (G.S.) combined the scores of the two raters and adjudicated “Velcro-type” crackles as present when the physicians disagreed.

### Correlation of acoustic and radiologic data

The relationships between “Velcro-type” crackles and individual features of pulmonary fibrosis were investigated by matching each recording with the corresponding HRCT section independently.

In order to determine the relationships between lung sounds and patterns on full volume HRCT, “Velcro-type” crackles were deemed as unilateral or bilateral in the individual patients according to their presence in the recordings from either one side or both sides of the chest, while they were considered absent when they could not be detected in any of the recordings from the same patient.

### Statistical analysis

The collected data was entered into the SPSS software package (version 24, IBM, USA) for statistical analysis. Continuous and categorical data were summarized using means and standard deviations, or counts and percentages respectively. Nominal or ordinal data were contrasted using a Chi-squared test. The inter-rater agreement was calculated using weighted Cohen’s kappa statistic (k_w_), and categorized as follows: poor (0 < k_w_ < 0.19), fair (0.20 < k_w_ < 0.39), moderate (0.40 < k_w_ < 0.59), good (0.60 < k_w_ < 0.79), and optimal (k_w_ > 0.81) [16]. Univariate and multivariate logistic regression was used to estimate the relationships between the individual radiologic features and patterns (independent variables) and audible “Velcro-type” crackles (dependent variable). For all analyses, statistical significance was set at *p* < 0.005. Where applicable, models were adjusted for the number of images per patient, since not all subjects were represented by the same number of HRCT images and sound files in the data set.

## Results

### Characteristics of study groups

On full HRCT scan review, 66 subjects (44.6%) had radiologic findings consistent with FILD. 10 subjects (6.7%) who did not meet radiological criteria for FILD on full HRCT review had evidence of isolated pulmonary fibrosis on single HRCT sections. Demographic characteristics of the groups defined according to presence of FILD on full volume HRCT are reported in Table 1. FILD and non FILD subjects had a mean age (SD) of 71 (8.2) and 67.55 (8.96) years respectively. Subjects in both groups were predominantly males (65.2 and 54.9%, respectively) and had more frequently a positive smoking history (56.1 and 68.3%). While FILD subjects had been initially referred to HRCT mostly for suspect of ILD (69.7%), half (52.4%) of non FILD subjects were sent to HRCT for other reasons, including general symptoms such as dyspnoea and cough, chronic obstructive pulmonary disease, follow-up of lung nodules, or haemoptysis. Among subjects with FILD, 15 (22.7%) and 31 (47%) had a definite or a possible UIP pattern at HRCT, respectively; in the remaining 20 (30.3%) patients with FILD the pattern was not consistent with UIP.

**Table 1:** Characteristics of study population. Data are expressed as counts (%) or mean with standard deviation (SD). FILD = Fibrotic Interstitial Lung Disease

	FILD (*n* = 66)	Non FILD (*n* = 82)
Age, years (SD)	71 (8.2)	67.55 (8.96)
Sex (%)		
Male	43 (65.2%)	45 (54.9%)
Female	23 (34.8%)	37 (45.1%)
Smoking history (%)		
Current/Former	37 (56.1%)	56 (68.3%)
Never smoker	29 (43.9%)	26 (31.7%)
Indication for HRCT (%)		
ILD	46 (69.7%)	19(23.2%)
Other	14 (21.2%)	43 (52.4%)
Unknown	6 (9.1%)	20 (24.4%)
Family History (%)		
Pulmonary Fibrosis	3 (4.5%)	1 (1.2%)
Autoimmune disease	13 (19.7%)	10 (12.2%)
HRCT pattern (%)		
UIP	15 (22.7%)	N/A
Possible UIP	31(47.0%)	
Inconsistent with UIP	20 (30.3%)	

### Inter-rater agreement

On single HRCT sections, the inter-observer agreement between the thoracic radiologists was good for the qualitative evaluation of fibrosis (k_w_ = 0.69, 95% CI 0.65–0.73). Among the individual radiologic features, the level of agreement was good for honeycombing (k_w_ = 0.71, 95% CI 0.63–0.79) and reticular opacities (k_w_ 0.65, 95% CI 0.62–0.68), while it was moderate for traction bronchiectasis (K_w_ = 0.51, 95% CI 0.47–0.55), fair for emphysema (K_w_ = 0.38, 95% CI 0.31–0.44) and poor for ground glass opacities (K_w_ = 0.28, 95% CI 0.22–0.34). The level of agreement between chest physicians as to the presence of bilateral “Velcro-type” crackles was good (k_w_ = 0.69, 95% CI 0.57–0.82).

### Relationships between “Velcro-type” crackles and patterns on full volume HRCT

The vast majority of subjects (81.1%) with “Velcro-type” crackles bilaterally on chest auscultation had a pattern consistent with FILD on subsequent HRCT scan. In contrast, FILD was present in only 26.8% of patients with unilateral “Velcro-type” crackles. Notably, 13 subjects (19.7%) with FILD on HRCT scan did not present “Velcro-type” crackles on chest auscultation. Of these, only one patient had a UIP pattern on HRCT, eight patients had possible UIP and four patients had a pattern inconsistent with UIP. On logistic regression analysis, the presence of bilateral “Velcro-type” crackles strongly predicted an FILD pattern (OR 13.46, 95% CI 5.85–30.96, *p* < 0.001), whilst unilateral “Velcro-crackles” were not significantly correlated (OR 0.58, CI 95% 0.29–1.16, *p* = 0.2) (Table 2). Different FILD patterns were independently associated with the presence of bilateral “Velcro-type” crackles on multivariate analysis, with the definite UIP pattern showing the strongest correlation (UIP: OR 19.8, 95% CI 5.28–74.25, *p* < 0.001; possible UIP: OR 13.09, 95% CI 4.87–35.2, p < 0.001; inconsistent with UIP: OR 10.8, 95% CI 3.85–32.85, p < 0.001).

**Table 2:** Relationships between presence of unilateral or bilateral “Velcro-type” crackles and radiologic pattern on HRCT. Data expressed as Odds ratio (OR) with 95% Confidence Intervals (95% CI) and *p* value

HRCT pattern	Bilateral “Velcro-type” crackles		Unilateral “Velcro-type” crackles	
	OR (CI 95%)	*p*	OR (CI 95%)	*p*
FILD	13.46 (5.71–29.182)	< 0.001	0.58 (0.29–1.16)	0.12
Definite UIP	19.8 (5.28–74.25)	< 0.001	0.49 (0.14–1.66)	0.25
Possible UIP	13.09 (4.87–35.2)	< 0.001	0.55 (0.23–1.34)	0.19
Inconsistent with UIP	10.8 (3.85–32.85)	< 0.001	0.75 (0.26–2)	0.53

### Relationships between “Velcro-type” crackles and HRCT features

On univariate regression analysis of 805 images and corresponding sound files, the presence of “Velcro-type” crackles predicted the presence of signs of pulmonary fibrosis (Table 3). All the individual features of FILD on HRCT showed a significant association with “Velcro-type” sounds, with traction bronchiectasis showing the strongest correlation (OR 4.37, 95% CI 3.17–6.02, *p* < 0.001) (Table 3A). Multivariate regression analysis was performed in order to assess the independent relationship between the extent of individual radiologic features and the presence of “Velcro-type” crackles (Table 3B). Reticulation had the strongest relationship (OR 2.04, 95% CI 1.62–2.57, *p* < 0.001), followed by honeycombing (1.88, 95% CI 1.24–2.83, *p* < 0.01), ground glass opacities (OR 1.74, 95% CI 1.29–2.32, *p* < 0.001) and traction bronchiectasis (OR 1.55, 95% CI 1.03–2.32, *p* < 0.05).

**Table 3:** Univariate (A) and multivariate (B) logistic regression of individual radiologic features on HRCT sections toward presence of “Velcro-type” crackles on corresponding recording sites. Data presented as odds ratios (OR) with 95% confidence intervals (CI) and *p* value

Feature	OR (95% CI)	*p* value
A		
Fibrosis	6.24 (4.5–8-66)	< 0.001
Ground glass opacities	2.13 (1.61–2.81)	< 0.001
Reticulation	2.57 (2.14–3.09)	< 0.001
Traction bronchiectasis	4.37 (3.17–6.02)	< 0.001
Honeycombing	2.39 (1.52–3.76)	< 0.001
Emphysema	0.72 (0.5–1.04)	0.077
B		
Ground glass opacities	1.74 (1.29–2.32)	< 0.001
Reticulation	2.04 (1.62–2.57)	< 0.001
Traction bronchiectasis	1.55 (1.03–2.32)	< 0.05
Honeycombing	1.88 (1.24–2.83)	< 0.01

Emphysema had a trend towards a negative association with the presence of “Velcro-type” crackles (OR 0.72, CI 95% 0.5–1.04, *p* = 0.077). To further investigate whether emphysema influences the transmission of lung sounds on chest auscultation in patients with coexisting fibrosis the mean scores for emphysematous alterations on single HRCT sections were compared between subgroups of FILD subjects with either bilateral, unilateral or absence of crackles on chest auscultation. Higher scores were found in patients with unilateral crackles (mean 0.3, SD 0.67) as compared to patients with bilateral or without crackles (mean 0.06, SD 0.23 and 0.11, SD 0.38), suggesting that in FILD subjects no clear link existed between the extent of emphysema and the absence of “Velcro-type” crackles.

## Discussion

We have performed the first prospective, blinded study to investigate the relationship between “Velcro-type” crackles and radiologic patterns and features of FILD. We showed that bilateral crackles correlate with the presence of FILD, and most strongly predict the presence of a UIP pattern at HRCT. “Velcro-type” crackles correlated with the extent of specific interstitial abnormalities in the lung parenchyma underneath. Features representing advanced structural fibrotic alteration (such as honeycombing) and also those suggesting less advanced fibrosis (i.e. a range of extent of reticular and ground glass opacities) were independently associated with the presence of “Velcro-type” crackles. Our finding that early radiologic signs of pulmonary fibrosis such as ground glass change and reticulation generate “Velcro-type” crackles supports the concept that lung sounds could provide a tool for the early identification of FILD.

Corroborating a previous report on the association of “Velcro-type” crackles on standard chest auscultation and ILD patterns on HRCT [17], our unbiased approach highlight the importance of bilateral “Velcro-type” crackles in triggering further investigation, specifically chest HRCT, in patients presenting with chronic respiratory symptoms. Furthermore, the finding that bilateral “Velcro-type” crackles were most strongly associated with the UIP pattern support the international consensus guideline recommendation to consider IPF in patients presenting with bibasilar crackles. On the other hand, the finding that crackles were not heard in nearly 20% of patients with FILD would apparently disprove the utility of subjective chest auscultation as a screening tool. Nevertheless, among these patients only one had UIP on HRCT, suggesting that crackles are hardly missed when honeycombing – the hallmark of UIP and therefore IPF - is present. Having said that, it must be pointed out that this study was not designed for diagnostic testing purposes, and any conclusion regarding the yield of “velcro-type” crackles’ assessment should not be drawn.

On individual HRCT sections the presence of pulmonary fibrosis was strongly associated with “Velcro-type” crackles. Extending this finding, multivariate analysis of individual radiologic features identified that reticulation, honeycombing, ground glass opacities and traction bronchiectasis were all independently associated with the presence of “Velcro-type” crackles in the lung parenchyma underneath. According to the stress-relaxation quadrupoles hypothesis developed through modeling by Fredberg in 1983 [18], crackles result from the acoustic energy produced by a change in elastic stress after a sudden opening or closing of distal airways. However, the pathological abnormalities underlying the generation of “Velcro-type” crackles and their pathologic significance have not been fully explored. The reticular pattern represents the hallmark of fibrotic interstitial lung disease at HRCT, correlating with a range of alterations from interlobular or intralobular thickening of the interstitial septa to the cyst walls of honeycombing [12]. The finding that the extent of reticular opacities is independently and strongly associated with “Velcro-type” crackles suggests that any degree of abnormal deposition of fibrotic tissue might cause the collapse of distal airways, according to the original theory of crackles generation [18]. Honeycombing is a term used to indicate the appearance of destroyed lung parenchyma and late stage fibrosis, presenting as piled, thick-walled cysts with a predominant distribution in the subpleural regions of the lungs. Assuming that airflow is maintained in these regions, it is likely that these dilated, distorted airspaces collapse during expiration and reopen during inspiration, causing the generation of the crackles. Ground glass opacities are areas of increased radiologic attenuation with preservation of bronchial and vascular margins, which may arise from partial filling of airspaces, increased capillary volume or interstitial intralobular thickening due to fluids or fibrosis [12]. The positive, independent association with “Velcro-type” crackles in this study strengthen the idea that even early stage interstitial involvement can be identified on chest auscultation. Traction bronchiectasis and bronchiolectasis represent abnormal bronchial and bronchiolar dilatation caused by surrounding fibrosis exerting a retractile force [12]. In the most peripheral regions, bronchiolectasis concurs to the architectural distortion of the lung parenchyma and may be actually seen as multiple cysts or microcysts, sometimes resembling honeycombing. As such, “Velcro-type” crackles might generate from these alterations as well following the same mechanisms.

Emphysema did not correlate with the presence of crackles, in keeping with the concept that destruction of alveolar walls result in a weaker transmission of lung sounds, either normal or adventitious [12]. In patients with coexisting fibrosis and emphysema it is possible that emphysema could mask “Velcro-type” crackles, thus decreasing the sensitivity of an acoustic assessment for fibrosis. To investigate this possibility, within this study we compared the emphysema scores between subgroups of FILD patients with different acoustic findings, and identified no evidence that emphysema reduces the sensitivity of auscultation to identify lung fibrosis.

This study has some points of strength. We recruited a large population of patients with a broad spectrum of lung pathologies. Lung sounds were recorded using a simple, point-of-care tool: an electronic stethoscope which is easily applicable in every clinical setting. The assessment of the recordings was performed by physicians blinded to any clinical information and unbiased towards their source (same or different patients). Lung sounds were paired with single HRCT slices: this approach allowed a precise matching with radiologic abnormalities in the lung parenchyma below the site of auscultation. Whilst lung sounds generated from a specific area might spread towards different regions of the same lung or to the other side of the chest, the transmission of “Velcro-type” crackles over different areas has been shown to be more limited than in other conditions such as chronic heart failure or pneumonia, supporting the rationale for the approach followed in this study [8]. The limitations of this study are mainly related to the research setting and cross-sectional design, which did not allow a comprehensive characterisation of the study population. The participants were not followed up and the final clinical diagnosis remained unknown in most cases. Physiology measurements and other clinical data were not collected, thereafter the relationships between “Velcro-type” crackles and clinical or functional deterioration were not addressed. Nevertheless, the study primarily focused on the validity of the subjective assessment of “Velcro-type” crackles against HRCT imaging, irrespectively of the clinical diagnosis or the functional status of the patients.

In conclusion, we identify that “Velcro-type” crackles not only predict the presence of FILD patterns at HRCT, but are also closely associated to the extent of different interstitial abnormalities in the lung parenchyma. Our finding that individual features of pulmonary fibrosis such as ground glass change and reticulation generate “Velcro-type” crackles warrants further investigation of the role of lung sounds as an early identification tool in FILD. The clinical utility of chest auscultation for assisting diagnosis and clinical management of ILD has been historically hampered by the subjectivity of standard chest auscultation and the poor signal transmission of standard stethoscopes [19]; if electronic auscultation were combined with computerized methods for lung sounds analysis and classification, this cost-effective approach might lead to the definition of an “acoustic signature” of FILD for both diagnostic and prognostic purposes, and this should be a focus of future studies.

## Abbreviations

CORSA: Computerized Respiratory Sounds Analysis
FILD: Fibrotic Interstitial Lung Disease
HRCT: High Resolution Computed Tomography
IPF: Idiopathic pulmonary fibrosis
UIP: Usual Interstitial Pneumonia
